# Using Monte Carlo methods for *H*_p_(0.07) values assessment during the handling of ^18^F-FDG

**DOI:** 10.1007/s00411-020-00864-9

**Published:** 2020-07-29

**Authors:** Łukasz Albiniak, Małgorzata Wrzesień

**Affiliations:** Department of Nuclear Physics and Radiation Safety, Faculty of Physics and Applied Informatics, University of Lodz, Pomorska 149/153, 90-236 Lodz, Poland

**Keywords:** Monte Carlo, Personal dose equivalent, *H*_p_(0.07), Nuclear medicine, FDG, Simulation

## Abstract

The dose limit for the skin of the hand is typically converted to a surface of 1 cm^2^, which means that one needs to measure point doses in different places on the hand. However, the commonly used method of measuring doses on the hand, i.e., using a dosimetric ring including one or several thermoluminescent detectors worn at the base of a finger, is not adequate for manual procedures such as labeling or radiopharmaceutical injection. Consequently, the purpose of this study was to create and conduct a series of computer simulations that, by recreating the actual working conditions, would provide information on the values of ionizing radiation doses received by the most exposed parts of the hands of employees of radiopharmaceutical production facilities, as well as those of nurses during the injection of radiopharmaceuticals. The simulations were carried out using Monte Carlo radiation transport calculations. The *H*_p_(0.07) personal dose equivalent values obtained for the fingertips of the index and middle fingers of nursing staff and chemists were within the range limited by the minimum and maximum *H*_p_(0.07) values obtained as a result of dosimetric measurements carried out in diagnostic and production centers. Only in the case of the nurse’s fingertip, the simulated value of *H*_p_(0.07 slightly exceeded the measured maximum *H*_p_(0.07) value. The comparison of measured and simulated dose values showed that the largest differences in *H*_p_(0.07) values occurred at the thumb tip, and for ring finger and middle finger of some of the nurses investigated.

## Introduction

Nuclear medicine is an interdisciplinary field and, therefore, a rather unusual branch of medicine. For example, a wide range of radionuclides are used, with various physical half-lives, and multiple methods of their preparation are needed. Very often, manual preparation of radiopharmaceuticals means that personnel employed in the field of nuclear medicine can be exposed to extremely high doses of ionizing radiation, especially when it comes to hand exposure. Significant difficulties in assessing the magnitude of this exposure result from the fact that fingertips are the most exposed areas, which is a consequence of the highly non-homogeneous distribution of doses on the skin of the hands and fingers.

Because the dose limit for the skin is converted to 1 cm^2^ of skin surface, one needs to measure point doses in different places on the hand. The commonly used method of measuring doses on the hand, i.e., using a dosimetric ring containing one or several thermoluminescent detectors worn at the base of a finger, however, is not adequate for manual procedures such as labeling or radiopharmaceutical injection (Martin and Sutton 2015; Wrzesień and Olszewski 2018; Sherbini et al. 2011; Lecchi et al. 2016; Jankowski et al. 2002; Sæther et al. 2005; Smart 2004; Chiesa et al. 1997; Donadille et al. 2005; Vanhavere et al. 2006; Batchelor et al. 1991; Montgomery et al. 1999; Hastings et al. 1997; Wrzesień and Olszewski 2011; Carnicer et al. 2011; Wrzesień et al. 2008, 2016; Kubo and Mauricio 2014). Unfortunately, dosimetric literature data on medical personnel who work with ionizing radiation in nuclear medicine facilities using positron emission tomography (PET) is typically limited to cases, where the exposure of personnel performing ^18^F-FDG injection procedures to the patient or diagnostic procedures has been analyzed (Leide-Svegborn 2010, 2011; Dalianis et al. 2006; Sandouqa et al. 2011; Antic et al. 2014; Wrzesień and Napolska 2015). When planning radiological protection for employees, it would be worth having at least approximate information about the dose values obtained by the most exposed parts of the hand. To this end, computer simulations can be used to obtain this information.

Currently, there are nine centers in Poland that produce ^18^F-FDG, and at least 16 medical facilities equipped with PET-CT hybrid devices. Interestingly, recently a group of independent experts expressed the view, after considering the epidemiology of cancer in individual European countries, that there should be 28 centers in Poland, each carrying out approximately 2000 examinations/year (Królicki et al. 2011). Thus, it is expected that the number of such centers will increase in the future, in Poland, highlighting the need for predicting the personnel exposure before it occurs.

Over the past decade, standard diagnostic imaging has increasingly involved procedures that use the PET technique (Królicki et al. 2011; Piciu 2012), which has increased the need for radiopharmaceuticals, especially fluorine-18 in the form of ^18^F-FDG. In turn, this development has increased the number of staff working in radiopharmaceutical production. However, the increase in the number of the patients diagnosed with ^18^F-FDG affects the doses received by nurses during the injection.

The analysis carried out as part of the ORAMED project (Vanhavere et al. 2012) regarding the exposure of the hands of nuclear medicine personnel is an important voice in the discussion on radiation protection aspects of this professional group. However, that project focused on manual deoxyglucose labeling procedures using ^18^F. In contrast, in Poland the labeling process has been automated and manual operations involving fluorodeoxyglucose include only the handling of ^18^F-FDG activity for the purposes of quality control of the finished products (Wrzesień et al. 2016).

The automation of the production process of the ^18^F positron marker and the automatic process of labeling deoxyglucose with the produced ^18^F contribute to the optimization of radiation protection, because both the number of employees and the time of contact with the radioisotope is reduced to the necessary minimum. However, this does not mean the complete elimination of manual procedures that involve the radioisotope. In fact, there are still procedures in the ^18^F-FDG production process that require manual intervention by an employee, in particular the quality control of the radiopharmaceutical. This is an important production stage, whose speed and effectiveness of implementation determine the release of the prepared product for further stages of production, including radiopharmaceutical injection to the patient. The procedures to be performed as part of the quality control are specified in the European Pharmacopoeia (2017), together with a description of their proper implementation.

All manual procedures carried out by employees responsible for quality control, as well as nurses injecting the radiopharmaceutical, mean working with an open source of ionizing radiation, which is ^18^F-FDG located in a vial or syringe. This work is the main source of exposure for chemists responsible for the quality control of ^18^F-FDG, and nurses.

The activity of radiopharmaceutical that is used at the beginning of any quality control procedure or the average ^18^F-FDG activity (regulated by reference levels) (Atomic Law 2019; COUNCIL DIRECTIVE 2013/59/EURATOM 2013) administered to a single patient, as well as the relatively short physical half-life (110 min) of ^18^F are the (physical) parameters that affect the dose that the employee’s fingertips receive during the work with ^18^F.

Currently, simulation methods are used in many fields of science including nuclear medicine and other branches of medicine that use ionizing radiation. The benefits of simulations are especially obvious in cases of inhomogeneous radiation fields, where measurements can be difficult, time consuming or not feasible at all (Becker et al. 2011). As stated by Becker et al., “Different scenarios can be simulated and situations with highest exposures can be revealed” (Becker et al. 2011). Simulations using voxel phantoms or flexible mathematical phantoms are useful tools for studying hand exposure (Becker et al. 2008a, b, 2011, 2014; Blunck et al. 2009, 2011; Becker and Blunck 2011; Figueira et al. 2013).

The purpose of the present study was to perform a series of computer simulations using the GEANT4 library package. By recreating the actual working conditions this allowed to obtain information on the radiation doses that are received by the most exposed parts of the hands of employees of radiopharmaceuticals production centres, as well as by nurses during injection. Information on working conditions and physical parameters affecting the level of recorded doses was obtained by physical measurements, which allowed verification of the dose values obtained by simulation.

## Materials and methods

### Simulation part

The simulations were carried out using the Monte Carlo method (Andreo 1991; Konefał 2006). For this purpose, the GEANT4 toolkit version 4.10.2 (GEometry ANd Tracking) was used to simulate the interaction of particles with matter (Agostinelli et al. 2003). Individual components of the created computer simulation are presented in the following sections.

#### Simulation “world”

To create the “world”, the built-in G4Box function was used and a cube with side *a* = 100 cm was implemented. The world was filled with air using the G4LogicalVolume and G4_AIR functions.

#### Worker’s hand

The process of creating an employee’s hand was multi-stage and consisted of several steps. The first was the choice of the hand model. For this it was important to realize that dosimetry measurements suggested that the quality control procedure and radiopharmaceutical injection have the greatest impact on the dose to the fingertips of personnel (Wrzesień et al. 2016, 2018; Wrzesień 2018a, b). Taking into account the fact that 70% of employees performing the above-mentioned procedures are women, the dimensions of the female hand model were used to construct the hand model. Hand dimensions (i.e., length, width, thickness, girth and radius of the palms) were selected from the corresponding 95th percentiles. Required data were taken from the atlas of human measures (Gedliczka 2001).

Based on this a mathematical hand model was created using geometric solids. The pastern was represented by a cuboid with the dimensions of *a* = 3.2 cm, *b* = 8.8 cm, and *c* = 11.7 cm (Gedliczka 2001). Individual fingers were built using the G4ElipticalTube function. Each of the fingers was composed of two phalanges. Pasterns and phalanges were uniformly filled with G4_SKIN_ICRP material from the embedded material database available in the GEANT4 package.

#### Syringe model

**Fig. 1:**
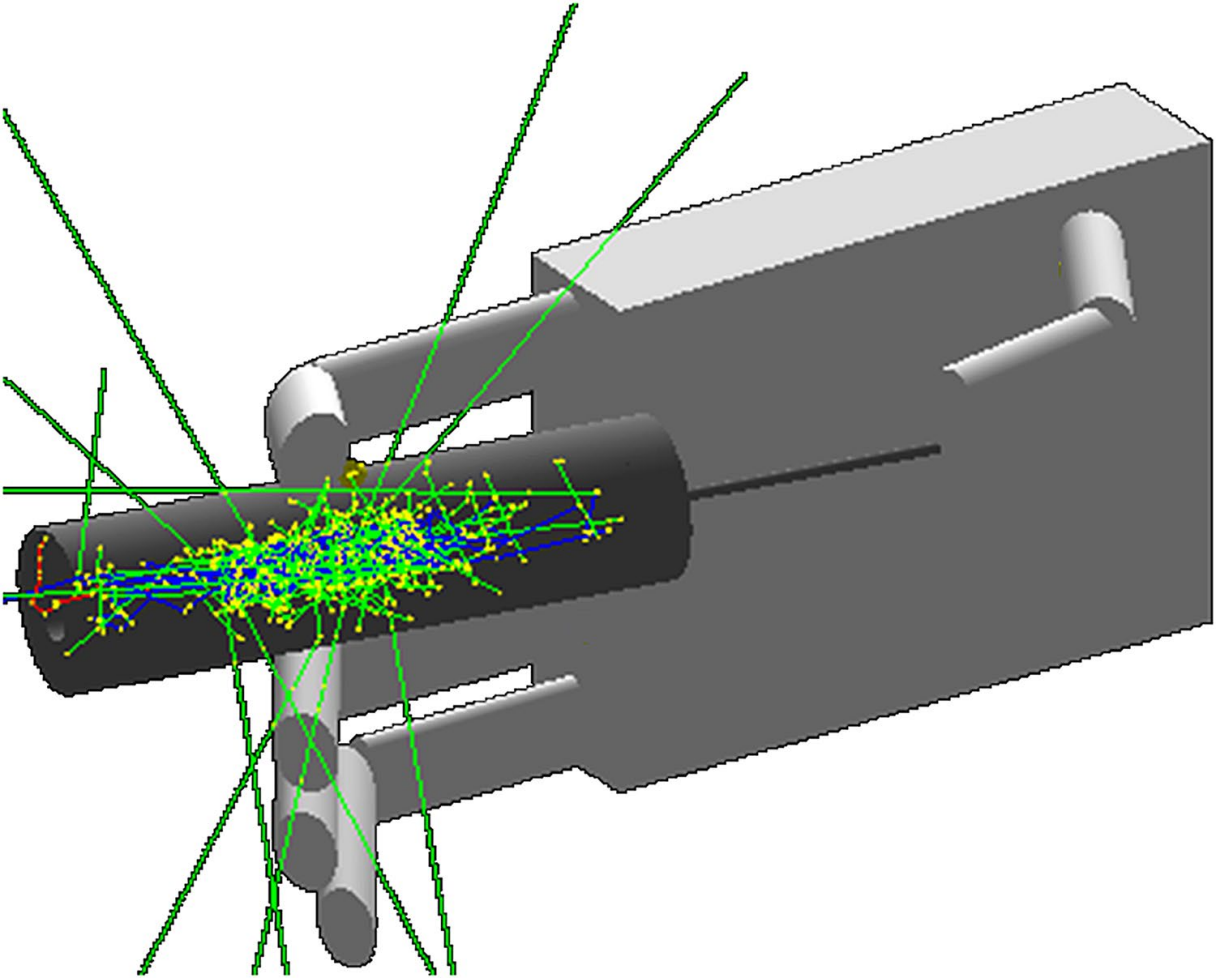
Developed model of syringe and dominant hand. Green tracks indicate gamma rays, red: electrons, blue: positrons. Yellow dots mark the places of interaction of radiation with matter (color figure online)

Quality control and radiopharmaceutical injection procedures are typically performed manually using a syringe. The activity accumulated in the syringe during manual operations, as well as the time needed to perform the appropriate procedure, determine the exposure of the hand to ionizing radiation, which is minimized by the use of an appropriate syringe cover. In the simulations a 9 mm tungsten shield was used. Figure 1 shows the developed syringe model and the dominant hand.

The syringe was modeled in the form of a hollow cylinder with an internal diameter of 10 mm (which simulates the inside of syringe), an external diameter of 29 mm (simulating the syringe shield), and a length of 100 mm. The plunger of the syringe was covered with a filled roller, 9 mm thick. Both the shield and roller were filled homogeneously with tungsten. The cylinder simulating the syringe plunger was filled homogeneously with air.

#### ^18^F radionuclide simulation in a syringe

Fluor-18 was created using the G4ParticleDefinition class. The ^18^F isotope was defined using corresponding mass and atomic numbers. The first atom was generated in the center of the syringe, and the next ones were created in a random position in relation to the originally created atom. Considering the processes of ^18^F decay, which involves creation of a positron with electron annihilation, and interaction of γ radiation with matter, required the use of various GEANT4 libraries: G4EmStandardPsychics, G4DecayPhysics and G4RadioactiveDecayPhysics. The neutrino resulting from the decay of the β^+^ atom ^18^F was eliminated from further processes, because it does not contribute to dose. This shortened the computer time needed for the simulation.

The ^18^F activity is determined by specifying the initial number of nuclei that undergo radioactive decay over time. In the present study, a typical average working time with an open source of radiation of 120 s was assumed, as well as an average activity with which the workers typically work of 300 MBq.

#### Simulation of doses

**Fig. 2:**
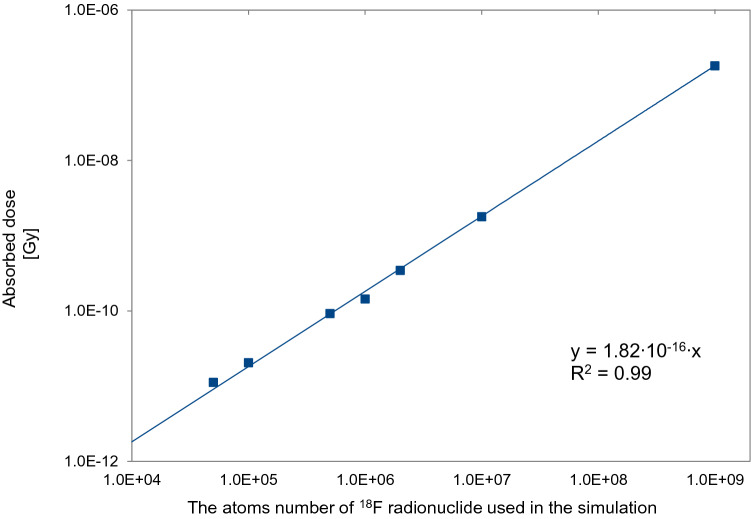
Relationship between the absorbed dose to the thumb and the number of ^18^F nuclei used in the simulation. Statistical uncertainties are smaller than the symbols shown

In principle, the simulations provided absorbed doses to the hand. At first, the simulations were carried out dozens of times for each of the given number of ^18^F nuclei of 100, 1000, 10,000, etc., respectively. The obtained values of absorbed doses for a given finger were averaged, and then the functional dependence of the absorbed dose and the number of ^18^F nuclei used in the simulation was plotted. Finally, the slope of this functional dependence was calculated for each finger considering that 3.6 × 10^10^ fluorine ^18^F nuclei correspond to an activity of 300 MBq. These slope values obtained (which provided absorbed dose to each finger per Bq of ^18^F activity) were used to calculate absorbed doses to each finger of the dominant hand, for any given radiopharmaceutical activity. Statistical uncertainties were smaller than the symbols shown in Fig. 2.

Comparison of measured with simulated doses required the conversion of the absorbed dose value into a personal dose equivalent *H*_p_(0.07) value [because dosemeters used for those dose measurements are typically calibrated in terms of *H*_p_(0.07)]. For this purpose, the radiation quality factor *Q*(*L*) = 1 for gamma radiation was used.

### Dosimetric measurements

To validate the results of the Monte Carlo simulations, *H*_p_(0.07) was measured in three PET-CT diagnostic facilities and in two facilities producing ^18^F-FDG radiopharmaceutical. Diagnostic centers are identified here as PET along with a number representing a certain facility. These facilities had also provided the data used in the present simulations, such as the activity of the radiopharmaceutical, the length of time an employee is typically exposed to ^18^F, and the technical data regarding the syringe. The measurements included highly sensitive MCP-N (LiF: Mg, Cu, P) thermoluminescence detectors manufactured by Radcard (Kraków, Poland). The detectors were calibrated in accordance with ISO 4037-3 (ISO 2019) as described in (Wrzesień et al. 2016, 2018; Wrzesień 2018a, b; Wrzesień and Napolska 2015). In total, 22 workers were included in the dosimetry measurements, including four physicists, nine chemists and nine nurses. The detectors were placed at 12 points of the palm side of both hands, namely on the fingertip of the thumb, index finger, middle finger, middle finger and small finger, and additionally at the base of the middle finger.

## Results

Figure 2 shows an example for the thumb of the relationship between the absorbed dose value and the number of ^18^F nuclei used in the simulation.

Analysis of the results obtained showed that for each of the modeled fingers, there exists a linear relationship between the number of atoms of the ^18^F radionuclide for which the simulations were carried out, and the value of the absorbed dose obtained. The *R*^2^ coefficients for the curves, determined for each of the fingers, were typically 0.99.

From these curves the slopes were obtained, for each of the five fingers.

Table 1 presents the values of these slopes for each of the fingers modeled in the simulation using the GEANT4 toolkit. These slope values allowed, taking into account the actual activity of the radiopharmaceutical, to calculate the value of absorbed doses for each of the five fingers of the dominant hand. The obtained results are presented in Table 1.

**Table 1:** Slope values for each curve obtained for the modeled fingers and absorbed dose values obtained from computer simulation for the fingers of the dominant hand

Finger	Slope (Gy/nuclei)	Absorbed dose obtained for 300 MBq of activity (mGy)
Thumb	1.82 × 10^−16^	0.006
Index	5.68 × 10^−15^	0.204
Middle	5.25 × 10^−15^	0.189
Ring	2.16 × 10^−15^	0.078
Small	1.07 × 10^−15^	0.039

## Discussion

To validate the dose values obtained by the MC simulations, results of dosimetric measurements obtained for one of the nurses from the PET I centre, for two nurses from the PET II centre, and for a chemist performing the quality control procedure at the RPC I centre were used. For these measurements, procedures selected during which the ^18^F activity in the employee’s syringe was about 300 MBq. Table 2 presents personal dose equivalent *H*_p_(0.07) values obtained by dosimetric measurements and computer simulations. The results presented in Table 2 lead to the following conclusions.

**Table 2:** Measured personal dose equivalent *H*_p_(0.07) values as compared to equivalent doses simulated with the Genat4 toolkit

Finger	H_*p*_(0.07) (mSv)				
	Simulation results	Nurse: PET I^a^	Nurse: PET II^b^	Nurse: PET II^b^	Chemist: quality control RPC I^c^
Thumb	0.006	0.044	0.052	0.078	0.015
Index	0.204	0.160	0.269	0.188	0.153
Middle	0.189	0.106	0.119	0.027	0.130
Ring	0.078	0.002	0.077	0.050	0.163
Small	0.039	0.064	0.079	0.060	0.056

^a^PET I: diagnostic centre I

^b^PET II: diagnostic centre II

^c^RPC I: radiopharmaceuticals production centre I

The simulations allow correct identification of the most exposed fingertips of the dominant hand of nurses injecting ^18^F-FDG and chemists performing radiopharmaceutical quality control. The *H*_p_(0.07) values obtained by simulation of the fingertips of index and middle fingers of nurses and chemists are within the limited range of minimum and maximum values obtained as a result of the dose measurements. Only in the case of the nurse’s ring fingertip, the *H*_p_(0.07) value obtained in the simulation slightly exceeds the maximum *H*_p_(0.07) value measured at PET II. The largest differences between measured and simulated *H*_p_(0.07) values were obtained for the thumb, for the ring finger of the nurse at PET I, and for the middle finger of the nurse at PET II.

In the case of the thumb, the observed difference between measured and simulated values can be explained by the change in position of the syringe plunger during the radiopharmaceutical injection procedure. The thumb presses on the plunger, forcing the radiopharmaceutical in the syringe into the patient’s vein. Note that in the simulation it was assumed that the piston of the syringe is stationary.

The second critical feature of computer model that affects the simulated results is the design of the syringe shield. For the GEANT4 simulations, both the body of the syringe and the part, where the plunger is located was assumed to be shielded. The implemented shield was a small cylinder filled with tungsten with a diameter corresponding to the diameter of the syringe, and with a thickness of several millimeters. The application of this shield resulted in the protection of the thumb against gamma radiation emitted from the side of the syringe piston. Such kind of are not used in production and diagnostic facilities. The results of the computer simulations can also be compared with dosimetric data published by other researchers. Table 3 summarizes the results of measurements regarding normalized values of *H*_p_(0.07) obtained during handling of ^18^F-FDG.

**Table 3:** Normalized *H*_p_(0.07) values obtained in this work as compared to those reported in the literature (Vanhavere et al. 2012; Wrzesień and Albiniak 2016; Carnicer et al. 2011; Covens et al. 2007)

Procedure	References	Maximum normalized dose (mSvGBq^−1^)		
		Range	Mean	Median
Handling of ^18^F-FDG (quality control procedure, injection procedure)	This work	0.13–0.68	0.35	0.29
Handling of ^18^F-FDG (quality control procedure of dispensing)	Wrzesień and Albiniak (2016)	0.02–0.85	0.50	0.35
^18^F preparation	Covens et al. (2007)	0.29–0.85	0.57	0.57
	Carnicer et al. (2011)	0.03–2.06	0.43	0.25
	Vanhavere et al. (2012)	0.10–4.43	1.20	0.83

## Conclusions

The simulations performed for assessment of the exposure of the dominant hand of nursing staff in PET centers and chemists in radiopharmaceutical production centers correctly predicted exposure to ionizing radiation resulting from the handling of a syringe containing ^18^F. Created based on the atlas of human measures, the mathematical hand model turned out to be a sufficient tool to assess exposure in the cases described in the present work. The personal dose equivalent *H*_p_(0.07) values to fingertips of employees obtained by application of the developed Monte Carlo method are mostly consistent with the results of dosimetric measurements carried out in PET diagnostic facilities and radiopharmaceuticals production centers. The actual dose values derived from these measurements, which depend on the radiopharmaceutical activity and the time needed to perform the required manual activities, proved valuable information about the doses for the most exposed parts of the employees’ hand.

The simulation algorithm created using the GEANT4 toolkit correctly identified the most exposed parts of the hand performing manual operations using a syringe containing ^18^F-FDG. The use of a ring dosimeter as part of routine measurements provides only partial information about fingertip exposure. In contrast, the use of computer simulations does allow the assessment of fingertip exposure and thus also the planning of radiation protection not only in the context of dose values but also in terms of the number of personnel carrying out manual procedures for radiopharmaceutical quality control and injections. Furthermore, changing the type of radionuclide that is the source of exposure in the simulation allows the method to be used in both conventional nuclear medicine facilities and centers producing short-lived radionuclides. Therefore, it seems reasonable to state that a properly constructed simulation can serve as a tool to support the work of radiological protection inspectors by predicting potential exposure in selected manual procedures and thus optimizing radiation protection before this exposure occurs.
